# Biological Activity and Chemical Composition of Propolis Extracts with Potential Use in Vulvovaginal Candidiasis Management

**DOI:** 10.3390/ijms25052478

**Published:** 2024-02-20

**Authors:** Ana Margarida Silva, Beatriz Rocha, Manuela M. Moreira, Cristina Delerue-Matos, José das Neves, Francisca Rodrigues

**Affiliations:** 1 REQUIMTE/LAQV, ISEP, Polytechnic of Porto, Rua Dr. António Bernardino de Almeida, 4249-015 Porto, Portugal; ana.silva@graq.isep.ipp.pt (A.M.S.); beasrocha@hotmail.com (B.R.); manuela.moreira@graq.isep.ipp.pt (M.M.M.); cmm@isep.ipp.pt (C.D.-M.); 2 i3S—Institute for Research and Innovation in Health, University of Porto, Rua Alfredo Allen, 208, 4200-135 Porto, Portugal; 3 INEB—Institute of Biomedical Engineering, University of Porto, Rua Alfredo Allen, 208, 4200-135 Porto, Portugal; 4 CESPU—Institute for Research and Advanced Training in Health Sciences and Technologies, Rua Central de Gandra 1317, 4585-116 Gandra, Portugal

**Keywords:** antioxidant activity, natural products, phenolic compounds, ultrasound-assisted extraction, vulvovaginal candidiasis

## Abstract

Environmental sustainability is an increasing challenge in the pharmaceutical field, leading to the search for eco-friendly active ingredients. Among natural ingredients, propolis arises as an excellent alternative, being a complex substance with pharmacological properties. This work aims to explore the potential of propolis as a new pharmaceutical ingredient for the replacement of conventional vulvovaginal antifungals. Propolis extracts were obtained by Ultrasound-Assisted Extraction using different solvents (water, water/ethanol (50:50, *v*/*v*), and ethanol). Afterwards, the extracts were characterized regarding total phenolic content (TPC), antioxidant/antiradical activities, radical scavenging capacity, antifungal activity against strains of Candida species, and viability effect on two female genital cell lines. The aqueous extract achieved the best TPC result as well as the highest antioxidant/antiradical activities and ability to capture reactive oxygen species. A total of 38 phenolic compounds were identified and quantified by HPLC, among which ferulic acid, phloridzin and myricetin predominated. Regarding the anti-*Candida* spp. activity, the aqueous and the hydroalcoholic extracts achieved the best outcomes (with MIC values ranging between 128 and 512 μg/mL). The cell viability assays confirmed that the aqueous extract presented mild selectivity, while the hydroalcoholic and alcoholic extracts showed higher toxicities. These results attest that propolis has a deep potential for vulvovaginal candidiasis management, supporting its economic valorization.

## 1. Introduction

The global population continues growing, being estimated to reach totals near 8.5 billion people by 2030 and 10.9 billion by 2100 [1]. A 30% increase in food supplies will be needed in comparison to the present, while major challenges are also expected to emerge in food security and agricultural practices [2]. Therefore, the search for sustainable development has generated an attempt to use natural matrices and products that are readily available, adding value to local resources and generating profits for small producers. At the same time, the use of plant and animal extracts in traditional medicine is one of the oldest human practices, particularly in developing countries where modern medicines are not always available or affordable [3]. Fruits and vegetables have long been described as excellent sources of polyphenols with demonstrated benefits for human health, such as antioxidant and cytoprotective activities [4]. Honey and related products, including propolis, are no exception [5]. Propolis is a natural adhesive resinous material produced by honeybees that results from the mixture of collected exudates of leaves, branches, and buds around the beehive with bee salivary secretions and beeswax. This complex matrix is used to build and seal cracks in the hive, protecting it from pathogens [6,7]. Propolis has a typically dark brown color, being solid and brittle at lower temperatures and becoming softer and stickier above 20 °C due to its resinous nature. Low toxicity and multiple functionalities also justify the traditional use of propolis for medical purposes [6]. Indeed, more than 300 constituents have been identified as bioactive [6,8]. Generally, propolis has been described as a rich source of benzoic acids and derivatives, cinnamic alcohol, cinnamic acid and respective derivatives, sesquiterpenes and triterpene hydrocarbons, benzaldehyde derivatives, alcohols, ketones, and heteroaromatic compounds, terpenes and sesquiterpene alcohols and their derivatives, aliphatic hydrocarbons, minerals, sterols and steroidal hydrocarbons, sugars, and amino acids [6,8,9]. Most important, the different combinations of these compounds are responsible for the antibacterial [10,11,12], antifungal [13,14], antiviral [15], anticancer [16,17], anti-inflammatory [18] and antioxidant [19,20,21] activities of propolis.

In particular, the ability of propolis to inhibit the growth of Candida species involved in vulvovaginal candidiasis (VVC) has recently attracted the attention of researchers [22,23,24,25,26]. VVC is typically caused by *Candida albicans* (around 90%), although cases of non-albicans VVC also occur and are usually more challenging to manage. Indeed, the failure of pharmacological treatments has increased due to intrinsic or acquired fungal resistance and can lead to cases of recurrent VVC (RVVC) [27]. This last condition affects 4% of women worldwide, causing genitourinary discomfort and inflammatory symptoms, and interfering with quality of life [28]. Hence, new alternative treatments are greatly required. Despite several previous reports on the potential application of propolis for the management of VVC [22,23,24,25,26], the use of the crude residue lacks the potential to yield products that could be used in the preparation of reproducible or even safe pharmaceutical products. Ultrasound-assisted extraction (UAE) arises as a green alternative extraction method, its main advantages being low-cost equipment and better extraction time as well as lower energy requirements [29]. This technique is based on cavitation, a phenomenon generated by the propagation of strong ultrasound waves in liquids [30] that cause the collapse of cavitation bubbles, leading to cell disruption and promoting a good penetration of the solvent into the cells and, consequently, to a better extraction of bioactive compounds [31]. Additionally, the probe system is more powerful than the ultrasound bath, producing additional energy and faster chemical reactions [30]. Therefore, the extraction of propolis bioactive compounds to manage vulvovaginal candidiasis could benefit from this sustainable technique that can be easily scaled up, constituting a more economical and eco-friendly alternative for industrial application [30]. To the best of our knowledge, this is the first study that employs UAE to obtain active ingredients from propolis crude residue.

In this work, eco-friendly UAE is employed to prepare different extracts of propolis obtained from the Natural Park of Montesinho, an area with protected designation of origin in northeast Portugal (Trás-os-Montes region). Extracts were screened and characterized in terms of phenolic composition, radical scavenging activity, antioxidant/antiradical properties, toxicity towards human genital cell lines, and biological activity against Candida species, aiming to select the best one to be used against VVC.

## 2. Results and Discussion

### 2.1. TPC and Antioxidant/Antiradical Activities

TPC is a spectrophotometric method widely used to evaluate the antioxidant activity of extracts from herbs, fruits, or cereals, among others [32,33,34,35]. The TPC results and antioxidant/antiradical activities of propolis extracts are summarized in Table 1.

The aqueous extract achieved the best result, followed by the hydroalcoholic and the alcoholic ones (217.7, 119.0, and 79.7 mg GAE/g dw, respectively). Significant differences were observed among all extracts (*p* < 0.01). According to Silva et al. [36], the TPC of propolis from Trás-os-Montes region ranged between 72.2 mg GAE/g dw and 277.2 mg GAE/g dw. Interestingly, a lower concentration was observed for the aqueous extract (72.2 mg GAE/g dw) in contrast to the present study (217.7 mg GAE/g dw). This difference may be due to the extraction method employed by the authors, which consisted of palynological processing using water, methanol, or 80% ethanol/water (1/10, *v*/*v*) as solvents [36]. In another study, Campo et al. [20] reported that the phenolic content was influenced by the sample’s origin, achieving a lower value in propolis obtained from the northern part of Portugal, probably due to the different apicultural practices implemented by beekeepers [36].

The antioxidant activity of propolis extracts was assessed by the FRAP assay, while the antiradical activity was screened via the ABTS method (Table 1). The aqueous extract presented the highest antioxidant activity, achieving an IC_50_ value of 77.2 μg/mL, while the hydroalcoholic and the alcoholic extracts obtained IC_50_ values of 169.8 μg/mL and 284.3 μg/mL, respectively, with significant differences being observed between all extracts (*p* < 0.05). Similarly, Lagouri et al. [37] studied the antioxidant activity of propolis collected from the Greek mainland (West Macedonia) and Rhodes (Greece), being both extracted with methanol, methanol 80% (*v*/*v*), and water, through conventional extraction procedures. The extract from West Macedonia prepared with methanol 80% obtained an IC_50_ value of 0.0065 mg/mL, while the aqueous extract from Rhodes reached an IC_50_ value of 0.1690 mg/mL [37], results considerably worse than the ones achieved in the present study.

Regarding antiradical activity, IC_50_ values ranged between 202.8 μg/mL and 469.7 μg/mL for the aqueous and alcoholic extracts, respectively (Table 1). Once again, significant differences were observed between the aqueous extract and the other extracts (*p* < 0.05), in contrast to the alcoholic and hydroalcoholic extracts (*p* = 0.349). Vongsak et al. [38] also analyzed the antiradical activity of propolis from three stingless bee species, *Lepidotrigona ventralis* Smith, *L. terminata* Smith, and *Tetragonula pagdeni* Schwarz, collected in Thailand. The extracts were prepared by sonication, with 80% of ethanol at 40 °C for 30 min, and, subsequently, with hexane at 40 °C for 20 min [38]. The ABTS assay led to IC_50_ values that varied between 59.5 and 605.4 μg/mL for *T. pagdeni* and *L. terminata*, respectively [38]. These results were in line with the ones obtained in the present study. As can be observed in Table 1, the aqueous extract presented the best results, followed by the hydroalcoholic and alcoholic extracts, which can be explained by the high polarity and affinity of water to the polar compounds. It should also be highlighted that the extraction yields for the aqueous, hydroalcoholic and alcoholic extracts were, respectively, 14.41 ± 0.71%, 24.63 ± 1.23%, and 48.66 ± 2.43%.

### 2.2. Identification and Quantification of the Phenolic Profile

**Figure 1:**
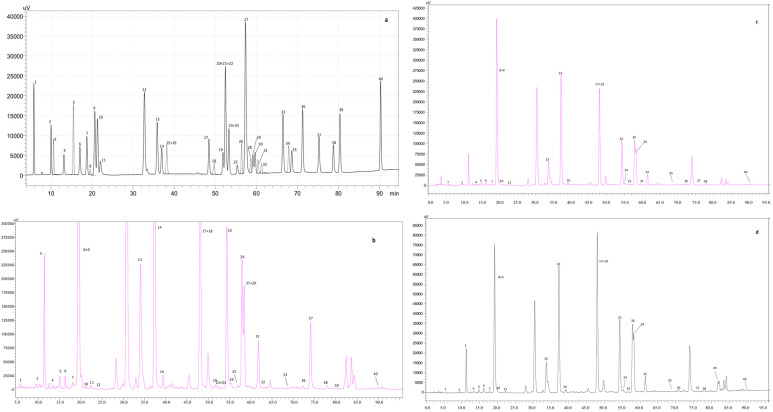
HPLC-DAD chromatograms at 280 nm for (**a**) polyphenols standard mixture of 5 mg/L, (**b**) propolis aqueous extract, (**c**) propolis hydroalcoholic (50:50; *v*/*v*) extract and (**d**) propolis alcoholic extract. Peak identification: (1) gallic acid, (2) protocatechuic acid, (3) neochlorogenic acid, (4) (+)-catechin, (5) caftaric acid, (6) caffeine, (7) chlorogenic acid, (8) 4-*O*-caffeyolquinic acid, (9) vanillic acid, (10) caffeic acid, (11) syringic acid, (12) (−)-epicatechin, (13) *p*-coumaric acid, (14) ferulic acid, (15) sinapic acid, (16) trans-polydatin, (17) naringin, (18) 3,5-di-caffeoylquinic acid, (19) quercetin-3-*O*-galactoside, (20) resveratrol, (21) quercetin-3-*O*-glucopyranoside, (22) rutin, (23) phloridzin, (24) ellagic acid, (25) 3,4-di-*O*-caffeoylquinic acid, (26) myricetin, (27) cinnamic acid, (28) quercitrin, (29) kaempferol-3-*O*-glucoside, (30) isorhamnetin-3-*O*-glucoside, (31) kaempferol-3-*O*-rutinoside, (32) isorhamnetin-3-*O*-rutinoside, (33) naringenin, (34) trans-epsilon viniferin, (35) quercetin, (36) phloretin, (37) tiliroside, (38) kaempferol, (39) apigenin, and (40) chrysin.

The identification of the phenolic compounds of the different extracts may justify the antioxidant and antiradical activities observed. A total of 38 compounds were identified in the extracts (Table 2). Figure 1 summarizes the chromatograms attained for the polyphenol’s standard mixture, as well as the aqueous, hydroalcoholic, and alcoholic extracts. In line with the results achieved for the spectrophotometric methods (Section 3.1), the aqueous extract showed the highest phenolic content.

The main compounds present in the three extracts were phenolic acids, mostly ferulic acid. Vanillic and *p*-coumaric acid were also quantified in considerable amounts in the aqueous extract, while *p*-coumaric acid and 3,5-di-caffeoylquinic acid were the most representative phenolics (after ferulic acid) in the hydroalcoholic and alcoholic extracts. Flavonols were the second major class of compounds present in hydroalcoholic and alcoholic extracts, representing 33.30% and 33.51%, respectively. Myricetin was the main flavonol quantified in all extracts, followed by phloridzin. Catechin was identified in all extracts, although epicatechin was only identified in the hydroalcoholic extract. Additionally, apigenin, a well-known flavone, was only identified in the alcoholic extract, while chrysin was present in all extracts.

The different extracts revealed high levels of flavonoids, in accordance with a previous report for European samples of propolis [39]. Ozkok et al. [40] evaluated the phenolic composition of propolis collected from different Turkish regions and reported the presence of six phenolic acids, namely caffeic acid, *p*-coumaric acid, trans-ferulic acid, protocatechuic acid, trans-cinnamic acid, and caffeic acid phenethyl ester. The authors also quantified flavonoids, such as quercetin (1.12–4.14 mg/g), galangin (0.72–40.79 mg/g), apigenin (1.07–17.35 mg/g), and pinocembrin (1.32–39.92 mg/g), although some of these compounds were not evaluated in this study. Lagouri et al. [37] analyzed the phenolic composition of Greek propolis and reported the presence of caffeic acid (0.64–4.17 mg/g), ferulic acid (0.53–1.41 mg/g), *p*-coumaric acid (0.83–3.00 mg/g), apigenin (0.48–2.74 mg/g), and galangin (1.32–8.55). Once again, the Portuguese propolis used in the present work seems to be richer in phenolic compounds when compared to the Greek propolis. These works demonstrate the richness of propolis in phenolic compounds and, most importantly, the influence of geographic conditions and the different extraction procedures on the bioactive composition of this complex matrix.

As expected, the phenolic profile results corroborate the antioxidant/antiradical results. The main phenolic compound quantified was ferulic acid, which is associated with antioxidant, antimicrobial, anti-inflammatory, anti-thrombosis, and anti-cancer properties [41]. In addition, the aqueous extract showed high amounts of vanillic acid when compared to the other extracts (2638 mg/100 g dw), which may justify the higher antiradical activity observed, being in line with previous authors [42]. Moreover, myricetin was also found in high quantities, acting as an antifungal against *C. albicans,* and reducing biofilm formation [43,44]. Catechin is another excellent antioxidant identified in the extracts. When used in combination with lower doses of antimycotics, catechin significantly inhibits the growth of fluconazole-resistant *C. albicans* [45]. These results highlight the antioxidant and anti-candidiasis effects of the phenolic compounds present in the propolis extracts prepared.

### 2.3. In Vitro Scavenging Capacity against ROS

ROS production is a consequence of normal metabolism, performing various physiological functions [46]. Although the role of moderately increased ROS levels in activating antifungal activity of neutrophils and macrophages may be beneficial for host response to vulvovaginal infection [47,48], the excessive production of these reactive species can exacerbate the inflammatory state associated with VVC [49]. The ROS scavenging capacities of the different extracts are summarized in Table 3.

Regarding the O_2_^•−^ uptake capacity, an IC_50_ value of 67.3 μg/mL was obtained for the aqueous extract, while a significantly higher value (651.4 μg/mL) was determined for the hydroalcoholic extract (*p* < 0.01). The scavenging capacity of the alcoholic extract was mild (inhibition percentage up to around 20%) at the highest tested concentration (1000 μg/mL). Furthermore, the aqueous extract attested a superior capacity to scavenge this oxygen species than the positive control catechin (IC_50_ = 84.4 μg/mL), supporting the excellent capacity of this extract.

Regarding the HOCl scavenging potential, the aqueous extract also achieved the highest activity (IC_50_ = 7.5 μg/mL), followed by the hydroalcoholic (IC_50_ = 11.3 μg/mL) and alcoholic extracts (IC_50_ = 38.1 μg/mL). Significant differences were observed (*p* < 0.05) for the alcoholic extract when compared to the other two, but not between the aqueous and the hydroalcoholic extracts (*p* = 0.130). Francisco et al. [50] registered IC_50_ values of 226.8 μg/mL and 13.3 μg/mL, respectively, for the scavenging activity against O_2_^•−^ and HOCl of the Brazilian propolis, highlighting the promising results for the extracts proposed in the present study. Additionally, the results are in line with the in vitro antioxidant/antiradical activities reported in the previous sections as well as the phenolic composition described. The higher scavenging efficacy of the aqueous propolis extract may be due to its superior content in phenolic compounds, particularly ferulic acid, well-known for its capacity to neutralize free radicals and act on the reduction of xanthine oxidase and cyclooxygenase activity [51]. Phloridzin is the main flavone found in propolis extracts and may inhibit the formation of O_2_^•−^ as well as lipid peroxides [52]. Flavonols, such as kaempferol, quercetin, and myricetin, have huge potential as ROS scavengers due to the number of hydroxyl groups on the B-ring [53,54]. Overall, the scavenging capacity of the aqueous and hydroalcoholic propolis extracts may be beneficial for the purpose of VVC management.

### 2.4. Antifungal Activity

The activity of the different extracts against six standard ATCC *Candida* spp. strains was determined according to the clinically relevant CLSI M27-A4 method (Table 4).

All extracts presented fungistatic activity, with MIC values varying from 128 to 512 μg/mL, with mild differences between extracts. The higher activity of the aqueous and hydroalcoholic extracts when compared to the alcoholic one may be related to their superior polyphenol content. Touzani et al. [55] also suggested that the antifungal activity of propolis is related to the presence of this type of compound. Values of MFC higher than 512 μg/mL for nearly all strains further reinforce the fungistatic nature of the tested extracts. Importantly, anti-Candida activity appeared to be maintained for strains resistant or featuring dose-dependent susceptibility to fluconazole, suggesting that the extracts could be useful in cases of azole-resistant VVC, particularly those with scarce availability of alternative treatment options [56]. According to Tobaldini-Valerio et al. [57], propolis extracts with MIC values < 800 μg/mL are potentially useful inhibitors of Candida spp. and considered suitable for topical therapy. Additionally, Duarte et al. [58] proposed a broader classification for plant products, stating that MIC values around 0.5 mg/mL indicate strong antifungal inhibitory activity. According to these authors, MIC values up to 2 mg/mL are still indicative of suitable activity for medical use. Thus, all tested extracts seem to be suitable as promising antifungal candidates for managing candidiasis.

### 2.5. Cytotoxicity Activity

The effect of propolis extracts on the viability of two relevant human cell lines of genital origin, viz. HEC-1-A and Ca Ski [59,60], was tested after 4 h of incubation. The relatively short time of exposure was selected to better mimic the typically brief residence time of drug products in the vagina [61]. The results are summarized in Table 5.

The mean CC_50_ values for the aqueous extract were above the maximum tested concentration (2048 μg/mL) in both cell lines and at least four times higher than the MIC values. These results suggest, at least, mild selectivity of the aqueous extract between host and pathogen cells. Moreover, the toxicity was higher for the hydroalcoholic and alcoholic extracts, with CC_50_ values of 896 μg/mL and 813 μg/mL in HEC-1-A cells and 1264 μg/mL and above 2048 μg/mL in Ca Ski cells, respectively.

Generally, lower CC_50_ values were reported by other authors for propolis extracts. For example, Banskota et al. [62] stated values from 51 μg/mL to over 100 μg/mL for different extracts of Brazilian propolis after 4 days of incubation with HT-1080 fibrosarcoma and murine colon 26-L5 cells. Bonamigo et al. [63] established CC_50_ values around 0.4-0.5 mg/mL for ethanolic extracts of propolis from *Apis mellifera* when tested in peripheral blood mononuclear cells and an erythroleukemia cell line upon 24 h of contact. Recently, Campoccia et al. [64] reported CC_50_ values for various poplar-type propolis extracts ranging from 70 to 85 μg/mL for MG63 osteosarcoma cells and lower than 40 μg/mL for L929 fibroblasts after overnight incubation. Therefore, the extracts prepared in the present study seem to be suitable for vaginal application, presenting low toxicity for both cell lines.

## 3. Materials and Methods

### 3.1. Chemicals

Gallic acid, sodium carbonate (Na_2_CO_3_), catechin, nitrotetrazolium blue chloride (NBT), ascorbic acid, 2,2′-azinobis-3-ethylbenzothiozoline-6-sulfonic (ABTS), potassium persulfate (K_2_S_2_O_8_), dihydrorodamine (DHR), and sodium hypochlorite (NaOCl) were purchased from Sigma-Aldrich, Taufkirchen, Germany. Ferric chloride (FeCl_3_), sodium hydroxide (NaOH), dimethylformamide (DMF), disodium (Na_2_HPO_4_), monopotassium phosphate (KH_2_PO_4_) phenol reagent appropriate for Folin-Ciocalteu (Folin) and Sabouraud dextrose broth (SDA) were obtained from Merck, Darmstadt, Germany. 2,4,6-Tris(2-pyridyl)-s-triazine (TPTZ), ferrous sulfate, β-nicotinamide adenine dinucleotide (NADH), and phenazine methosulfate (PMS) were purchased from Sigma-Aldrich, Buchs (Switzerland), Anekal Taluk (India), and Burlington, VT, (USA), respectively. Sodium acetate, RPMI 1640 and morpholinopropanesulfonic acid (MOPS) was sourced from Sigma Chemical Co., Burlington, VT, USA, acetic acid from Chem-Lab NV, Zedelgem, Belgium, and anhydrous absolute ethanol from Carlo Erba Reagents, Val-de-Reuil, France. Ultra-pure water was obtained in-house using a Milli-Q water purification system (TGI Pure Water Systems, San Diego, CA, USA). All other chemicals were of analytical grade or equivalent.

### 3.2. Propolis Samples and Extraction

Propolis was collected from *Apis mellifera* L. bee hives in apiaries located in the Natural Park of Montesinho (41°53′49″ N, 6°51′58″ W) in September 2021. The crude extracts were packed in sealed plastic bags and stored at −18 °C until further use. The propolis extraction was conducted by UAE using ethanol, water, or an hydroalcoholic mixture (50:50, *v*/*v*) as solvent, according to the procedure described by Cavalaro et al. [65]. Briefly, the extracts were obtained using an ultrasonic processor (Sonics Vibra-cell^TM^, VCX 500/VCX 750, Lutterworth, UK) with a frequency of 20 KHz and a probe (630–0220) with 13 mm diameter. For extraction, 0.86 g of sample was added to 30 mL of solvent for 20 min, at 25 °C and with 30% sonication amplitude. The samples were subsequently centrifuged at 4700× *g* for 15 min at 25 °C and filtered using Whatman no. 2 filters. The aqueous extracts were frozen at −80 °C until lyophilization (Telstar LyoQuest, Barcelona, Spain), while the alcoholic and hydroalcoholic extracts were kept under refrigeration at 4 °C until evaporation in a rotary evaporator (Vacuum Controller V-800, Büchi, Aesch, Switzerland) at 40 °C. The yield was calculated using the dry weight of the extract and soaked samples.

### 3.3. Determination of Total Phenolic Content

The total phenolic content (TPC) was calculated spectrophotometrically, based on a complex redox reaction, as described by Pinto et al. [66]. The reaction mixture occurred in each well of a 96-well microplate and consisted of a mixture of sample, Folin-Ciocalteu reagent, and Na_2_CO_3_ solution (7.5%, *w*/*v*). Samples were in a concentration of 500 µg/mL. The absorbance was read at 765 nm using a Synergy HT Microplate Reader (BioTek Instruments, Winooski, VT, USA). Gallic acid was used as standard (linearity range = 5–100 µg/mL; *R*^2^ = 0.9992). The results were expressed as milligrams of gallic acid equivalents (GAE) per gram of dry weight (dw) (mg GAE/g dw).

### 3.4. Determination of In Vitro Antioxidant/Antiradical Activities

#### 3.4.1. Ferric Reducing Antioxidant Power

The ferric ion reduction antioxidant capacity (FRAP) was calculated based on the reduction of a ferric 2,4,6-trypyridyl-s-triazine complex (Fe^2+^-TPTZ) to the ferrous form (Fe^3+^-TPTZ), as described by Benzie and Strain [67], with minor modifications. The assay was performed directly in a 96-well microplate, adding sample and FRAP reagent to each well. The reaction mixture was incubated at 37 °C for 30 min and the absorbance was read at 595 nm in a Synergy HT Microplate Reader. Ferrous sulfate 1 mM (FeSO_4_∙7H_2_O) was used as standard (linearity range: 25–500 μM; *R*^2^ = 0.9997). The results were presented as half-maximal inhibitory concentration (IC_50_) values.

#### 3.4.2. ABTS Radical Scavenging Assay

The evaluation of the ABTS radical sequestration capacity was performed according to the methodology described by Re et al. [68], with minor modifications. The assay was performed directly in a 96-well microplate by adding ABTS solution and sample to each well. Ascorbic acid was used as standard (linearity range: 5–100 μg/ mL; *R*^2^ > 0.9922). The results were expressed as IC_50_ values.

### 3.5. Phenolic Profile Analysis

Propolis extracts were analyzed by high performance liquid chromatography with a diode-array detector (HPLC-DAD), as described by Moreira et al. [69]. The chromatographic separation was carried out on a reversed-phase Phenomenex Gemini C18 column (250 × 4.6 mm, 5 µm particle size) at 25 °C. The mobile phase comprised methanol and water, both with 0.1% of formic acid, and a gradient program was used. The chromatograms were acquired at a wavelength of 280 nm by a photodiode array detector (Merck^®^ Hitachi Diode Array Detector L-2455, Kent, UK). The results were expressed as mg of each phenolic compound per 100 g of extract on dw (mg/100 g dw).

### 3.6. Assessment of Reactive Oxygen Species Scavenging Capacity

#### 3.6.1. Superoxide Radical Scavenging Assay

The superoxide anion radical (O_2_^•−^) scavenging capacity was determined spectrophotometrically, as described by Gomes et al. [70]. Absorbance was read at 560 nm for 6 min at 37 °C in a Synergy HT Microplate Reader. The results were expressed as IC_50_ values of the reduction of NBT to a purple-colored diformazan upon reaction with O_2_.

#### 3.6.2. Hypochlorous Acid Scavenging Assay

The uptake capacity of hypochlorous acid (HOCl) was determined by monitoring the effect of propolis on the HOCl-induced oxidation of DHR to rhodamine, according to Gomes et al. [70]. The fluorescence was read at 37 °C for 5 min, at wavelengths of 485 ± 20 nm and 528 ± 20 nm. The results were expressed as the inhibition (IC_50_ values) of HOCl-induced DHR oxidation.

### 3.7. Determination of Antifungal Activity

The activity of propolis extracts against *Candida* spp. was determined using the CLSI M27-A4 broth microdilution method [71]. Six reference strains from the American Type Culture Collection (ATCC, Manassas, VA, USA) were used, namely *C. albicans* (ATCC 90028 and ATCC 64550), *C. glabrata* (ATCC 2001), *C. parapsilosis* (ATCC 22019), *C. krusei* (ATCC 6258) and *C. tropicalis* (ATCC 750). In brief, isolates were subcultured on Sabouraud Dextrose Broth (SDA) for 24 h at 37 °C before being dispersed in RPMI 1640 medium supplemented with MOPS (pH = 7.0) to a final concentration of 0.5–2.5 × 10^3^ cells/mL. The assay was performed in 96-well microplates by mixing 100 µL of *Candida* spp. dispersions with 100 µL of extracts dispersed in the same medium. Final concentration of propolis extracts ranged from 4 to 512 µg/mL. The minimum inhibitory concentration (MIC; defined as the lowest concentration without growth) was determined after 48 h of incubation at 37 °C by visual inspection. Experiments were performed in triplicate. Additionally, the minimal fungicidal concentration (MFC) was assessed by collecting 20 µL of the content of wells at MIC and higher concentrations and plating it onto SDA in duplicate for 24 h at 37 °C. MFC values were defined as the lowest concentration at which no apparent growth was observable.

### 3.8. Cell Viability Assays

The toxicity of propolis extracts to human cell lines of female genital tract origin, namely HEC-1-A endometrial cells and Ca Ski cervical cells (ATCC), was determined through the resazurin reduction assay [72]. These cell lines were selected since they are representative in vitro models of the female genital epithelia and have been used in the past for screening the toxicity of drugs, excipients and formulations intended for vaginal use [73,74]. HEC-1-A cells and Ca Ski cells were maintained in McCoy’s 5A medium and RPMI 1640 medium, respectively, in both cases supplemented with 10% (*v*/*v*) fetal bovine serum, 100 U/mL penicillin and 100 μg/mL streptomycin, and kept at 37 °C, 95% relative humidity (RH), and 5% CO_2_. Cells were seeded at a density of 5000/well in 96-well plates and incubated for 24 h, after which propolis extracts were added at different concentrations (128–2048 μg/mL) and cells incubated for an additional 4 h. Cells incubated with plain culture medium and 1% (*w*/*v*) Triton X-100 were also used as controls. Resazurin was then added at a concentration of 10 μg/mL and cells left to incubate for 3 h. Finally, supernatants (100 μL) were transferred to an opaque 96-well plate and the fluorescence was measured at 590/530 nm using a Synergy HT Multi-Mode plate reader (BioTek). Experiments were performed in triplicate and cell viability was used to calculate half-maximal cytotoxic concentration (CC_50_) values by log-logistic regression using Prism 8 (Graph-Pad, La Jolla, CA, USA).

### 3.9. Statistical Analysis

The results are presented as mean ± standard deviation (*n* = 3). Microsoft Office Excel 2020 and SPSS Statistics 28.0 software were used for data analysis. One-way ANOVA test, followed by HSD Tukey’s post-hoc test, was applied to assess differences between trials. A paired sample Student’s t-test was also performed to compare the means of the variables with each other. Values of *p* < 0.05 were considered as statistically significant.

## 4. Conclusions

The present work reported for the first time the assessment of propolis from the Natural Park of Montesinho, a protected Portuguese region, as a potential new antifungal ingredient for pharmaceutical applications. The green extraction methodology employed, coupled with the sustainable solvents used, allowed us to obtain extracts rich in bioactive compounds, with considerable antioxidant and antiradical activities. The aqueous extract achieved the best outcomes in the spectrophotometric tests employed, exhibiting a phenolic profile mainly characterized by the presence of ferulic acid, vanillic acid, *p*-coumaric acid, and myricetin. Also, this extract was shown to be effective against the tested Candida species. The cell viability assay attested the low toxicity of the aqueous extract in both cell lines used (Ca Ski and HEC-1-A). Therefore, the aqueous extract was revealed to be the most promising, presenting antioxidant and anti-candidiasis effects commonly involved in VVC. In the future, complementary studies, such as in vitro permeability assays, should be performed to ensure the safety and efficacy of this extract against VVC. Moreover, to complement the anti-fungal activity, the antimicrobial capacity should be analyzed.

## Figures and Tables

**Table 1:** Total phenolic content (TPC) and antioxidant/antiradical activities of propolis extracts based on their abilities to reduce ferric iron (Fe^3+^) to ferrous iron (Fe^2+^) and to sequester the ABTS radical. Results are expressed as mean ± standard deviation (*n* = 3). Different letters (a, b, c) in the same column indicate significant differences between mean values (*p* < 0.05).

Propolis Extracts	TPC	FRAP	ABTS
	mg GAE/g dw	IC_50_ (μg/mL)	IC_50_ (μg/mL)
Aqueous	217.7 ± 5.1 ^a^	77.2 ± 2.1 ^a^	202.8 ± 14.9 ^a^
Hydroalcoholic	119.0 ± 5.3 ^b^	169.8 ± 4.4 ^b^	463.1 ± 39.6 ^b^
Alcoholic	79.7 ± 3.8 ^c^	284.3 ± 6.7 ^c^	469.7 ± 33.9 ^b^

**Table 2:** Phenolic compounds identified and quantified in propolis extracts through HPLC-DAD analysis (*n* = 3). Results are expressed as mean ± standard deviations (mg of phenolic compound/100 g dw).

Phenolic Compound	Aqueous	Hydroalcoholic	Alcoholic
	(mg/100 g dw)	(mg/100 g dw)	(mg/100 g dw)
Phenolic acids			
Gallic acid	34.1 ± 1.7	26.6 ± 1.3	13.9 ± 0.7
Protocatechuic acid	74.0 ± 3.7	17.1 ± 0.9	<LOD
Neochlorogenic acid	49.1 ± 2.5	<LOD	6.1 ± 0.3
Caftaric acid	26.3 ± 1.3	27.1 ± 1.4	9.5 ± 0.5
Chlorogenic acid	168 ± 8	27.6 ± 1.4	22.0 ± 1.1
4-*O*-caffeyolquinic acid	293 ± 15	112 ± 6	44.4 ± 2.2
Vanillic acid	2638 ± 132	29.4 ± 1.5	7.1 ± 0.4
Caffeic acid	<LOQ	12.2 ± 0.6	6.4 ± 0.3
Syringic acid	26.6 ± 1.3	29.0 ± 1.4	14.4 ± 0.7
*p*-coumaric acid	767 ± 38	585 ± 29	256 ± 13
Ferulic acid	2833 ± 142	2193 ± 110	1081 ± 54
Sinapic acid	<LOQ	ND	ND
3,5-di-caffeoylquinic acid	507 ± 25	241 ± 12	113 ± 6
Ellagic acid	<LOD	60.9 ± 3.0	32.9 ± 1.6
3,4-di-*O*-caffeoylquinic acid	20.9 ± 1.0	<LOQ	18.8 ± 0.9
Cinnamic acid	215 ± 11	ND	ND
Σ Phenolic acids	7652.0 ± 382.5	3360.9 ± 168.5	1625.5 ± 81.7
Flavanols			
(+)-Catechin	95.0 ± 4.7	72.4 ± 3.6	19.6 ± 1.0
(−)-Epicatechin	<LOQ	16.6 ± 0.8	ND
Σ Flavanols	95.0 ± 4.7	89.0 ± 4.4	19.6 ± 1.0
Flavanones			
Naringin	35.7 ± 1.8	529 ± 26	249 ± 12
Naringenin	9.0 ± 0.5	<LOQ	<LOD
Σ Flavanones	44.7 ± 2.3	529.0 ± 26.0	249.0 ± 12.0
Flavonols			
Quercetin-3-*O*-galactoside	17.3 ± 0.9	ND	ND
Quercetin-3-*O*-glucopyranoside	<LOQ	ND	ND
Rutin	<LOD	ND	ND
Myricetin	1783 ± 89	2839 ± 142	1444 ± 72
Kaempferol-3-*O*-glucoside	63.5 ± 3.2	39.5 ± 2.0	3.2 ± 0.2
Kaempferol-3-*O*-rutinoside	58.0 ± 2.9	22.2 ± 1.1	<LOD
Isorhamnetin-3-*O*-rutinoside	<LOD	40.1 ± 2.0	22.6 ± 1.1
Isorhamnetin-3-*O*-glucoside	ND	ND	ND
Quercetin	<LOQ	20.1 ± 1.0	17.2 ± 0.9
Quercitrin	ND	ND	ND
Tiliroside	55.4 ± 2.8	12.2 ± 0.6	3.0 ± 0.2
Kaempferol	24.6 ± 1.2	17.5 ± 0.9	13.0 ± 0.6
Σ Flavonols	2001.8 ± 100.0	2990.06 ± 149.6	1503.0 ± 75.0
Flavones			
Apigenin	<LOD	<LOQ	7.6 ± 0.4
Chrysin	10.9 ± 0.5	5.0 ± 0.3	2.9 ± 0.1
Σ Flavones	10.9 ± 0.5	5.0 ± 0.3	10.5 ± 0.5
Others			
Caffeine	75.9 ± 3.8	75.5 ± 3.8	12.3 ± 0.6
trans-polydatin	163 ± 8	54.0 ± 2.7	48.3 ± 2.4
Resveratrol	<LOQ	<LOQ	ND
Phloridzin	2036 ± 102	1834 ± 92	996 ± 50
Phloretin	24.6 ± 1.2	44.7 ± 2.2	20.0 ± 1.0
trans-epsilon viniferin	<LOD	<LOQ	ND
Σ Others	2299.5 ± 115.0	2008.2 ± 100.7	1076.6 ± 54.0

ND: not detected; LOD: limit of detection; LOQ: limit of quantification.

**Table 3:** O_2_^•−^ and HOCl scavenging capacities of propolis extracts. Values are expressed as mean ± standard deviation (*n* = 3). Different letters (a, b) in the same column indicate significant differences between mean values (*p* < 0.05).

	ROS	
	O_2_^•−^	HOCl
	IC_50_ (μg/mL)	
Propolis extracts		
Aqueous	67.3 ± 1.0 ^a^	7.5 ± 1.2 ^a^
Hydroalcoholic	651.4 ± 11.2 ^b^	11.3 ± 0.8 ^a^
Alcoholic	>1000	38.1 ± 3.9 ^b^
Positive Controls		
Gallic acid	24.6 ± 1.5	0.7 ± 0.1
Catechin	84.4 ± 4.6	0.1 ± 0.01

**Table 4:** Anti-Candida activity of propolis extracts. Results are presented as MIC and MFC values against ATCC strains and vaginal isolates of Candida species. Results are presented singly or as range values (*n* = 3). Sensitivity of each strain to fluconazole is presented for reference purposes.

**Strains**	**Fluconazole Susceptibility**	**Aqueous**		**Hydroalcoholic**		**Alcoholic**	
		**MIC** **(μg/mL)**	**MIC** **(μg/mL)**	**MFC** **(μg/mL)**	**MFC** **(μg/mL)**	**MIC** **(μg/mL)**	**MFC** **(μg/mL)**
*C. albicans* ATCC 90028	S	256	128–256	>512	>512	512	>512
*C. albicans* ATCC 64550	R	128–256	128–512	>512	>512	512	>512
*C. glabrata* ATCC 2001	S-DD	≈512	≈512	>512	>512	≈512	>512
*C. parapsilosis* ATCC 22019	S	256	128	512	>512	512	>512
*C. krusei* ATCC 6258	S-DD	512	256–512	>512	>512	≈512	>512
*C. tropicalis* ATCC 750	S	≈512	256–512	>512	>512	≈512	>512

S: susceptible; S-DD: susceptible (dose-dependent); R: resistant.

**Table 5:** Effects of propolis extract exposure on the viability (%) of HEC-1-A and Ca Ski cell lines at different concentrations, as measured by the resazurin assay. Values are expressed as mean ± standard deviation (*n* = 3). Different letters (a, b, c, d) in the same line indicate significant differences between mean values (*p* < 0.05).

Sample			Concentration (μg/mL)		
	128	256	512	1024	2048
			HEC-1-A		
**Aqueous extract**	102.70 ± 5.14 ^a^	102.50 ± 6.01 ^a^	59.30 ± 2.97 ^b^	56.60 ± 5.83 ^b^	66.11 ± 8.06 ^b^
**Hydroalcoholic extract**	91.80 ± 4.00 ^a^	90.70 ± 6.80 ^a^	60.40 ± 7.80 ^b^	33.60 ± 1.70 ^c^	36.60 ± 1.80 ^c^
**Alcoholic extract**	121.80 ± 5.14 ^a^	78.50 ± 6.01 ^b^	69.90 ± 2.97 ^b^	34.80 ± 5.83 ^c^	7.30 ± 0.20 ^d^
**Positive control**	99.55 ± 4.09				
**Negative control**	0.00 ± 0.45				
			**Ca Ski**		
**Aqueous** **extract**	127.80 ± 5.14 ^a^	123.90 ± 6.01 ^a^	90.00 ± 2.97 ^b^	87.00 ± 5.83 ^b^	85.60 ± 8.06 ^b^
**Hydroalcoholic extract**	111.70 ± 6.63 ^a^	104.50 ± 6.05 ^a^	74.10 ± 2.20 ^b^	54.80 ± 0.10 ^c^	16.00 ± 0.81 ^d^
**Alcoholic extract**	112.60 ± 6.62 ^a^	114.10 ± 5.72 ^a^	108.50 ± 5.43 ^a^	59.50 ± 2.97 ^b^	26.60 ± 1.60 ^c^
**Positive control**	101.60 ± 6.08				
**Negative control**	0.00 ± 0.56				

## Data Availability

Data contained within the article.

## References

[1] United Nations, Department of Economic and Social Affairs, Population Division (2019). World Population Prospects 2019: Highlights.

[2] Wezel A., Casagrande M., Celette F., Jean-François V., Ferrer A., Peigné J. (2014). Agroecological practices for sustainable agriculture. A review. Agron. Sustain. Dev..

[3] Peter C., Waller S., Picoli T., da Gama Osório L., Zani J.L., Meireles M., Faria R.O., Mello J., Hubner S., Lima M. (2019). Chemical and cytotoxic analyses of three varieties of Brazilian propolis (green propolis, jataí propolis and brown propolis) and its anti-Sporothrix brasiliensis in vitro activity. Arq. Bras. Med. Vet..

[4] Kabir F., Tow W.W., Hamauzu Y., Katayama S., Tanaka S., Nakamura S. (2015). Antioxidant and cytoprotective activities of extracts prepared from fruit and vegetable wastes and by-products. Food Chem..

[5] FAO https://www.fao.org/fsnforum/consultation/beekeeping.

[6] Damodaran T., Gupta R.C., Lall R., Srivastava A. (2021). Chapter 46—Propolis. Nutraceuticals.

[7] Burdock G.A. (1998). Review of the biological properties and toxicity of bee propolis (propolis). Food Chem. Toxicol..

[8] Cauich-Kumul R., Segura Campos M.R., Campos M.R.S. (2019). Chapter 12—Bee Propolis: Properties, Chemical Composition, Applications, and Potential Health Effects. Bioactive Compounds.

[9] Anjum S.I., Ullah A., Khan K.A., Attaullah M., Khan H., Ali H., Bashir M.A., Tahir M., Ansari M.J., Ghramh H.A. (2019). Composition and functional properties of propolis (bee glue): A review. Saudi J. Biol. Sci..

[10] Okińczyc P., Paluch E., Franiczek R., Widelski J., Wojtanowski K.K., Mroczek T., Krzyżanowska B., Skalicka-Woźniak K., Sroka Z. (2020). Antimicrobial activity of *Apis mellifera* L. and *Trigona* sp. propolis from Nepal and its phytochemical analysis. Biomed. Pharmacother..

[11] Lu L.C., Chen Y.W., Chou C.C. (2005). Antibacterial activity of propolis against Staphylococcus aureus. Int. J. Food Microbiol..

[12] Marcucci M.C., Ferreres F., García-Viguera C., Bankova V.S., De Castro S.L., Dantas A.P., Valente P.H., Paulino N. (2001). Phenolic compounds from Brazilian propolis with pharmacological activities. J. Ethnopharmacol..

[13] Ożarowski M., Karpiński T.M., Alam R., Łochyńska M. (2022). Antifungal Properties of Chemically Defined Propolis from Various Geographical Regions. Microorganisms.

[14] Koç A., Silici S., Kasap F., Hörmet-Oz H., Mavus-Buldu H., Ercal B. (2011). Antifungal Activity of the Honeybee Products against *Candida* spp. and *Trichosporon* spp.. J. Med. Food.

[15] Mazia R.S., de Araújo Pereira R.R., de Francisco L.M.B., Natali M.R.M., Dias Filho B.P., Nakamura C.V., Bruschi M.L., Ueda-Nakamura T. (2016). Formulation and Evaluation of a Mucoadhesive Thermoresponsive System Containing Brazilian Green Propolis for the Treatment of Lesions Caused by Herpes Simplex Type I. J. Pharm. Sci..

[16] Banskota A.H., Nagaoka T., Sumioka L.Y., Tezuka Y., Awale S., Midorikawa K., Matsushige K., Kadota S. (2002). Antiproliferative activity of the Netherlands propolis and its active principles in cancer cell lines. J. Ethnopharmacol..

[17] Orsolić N., Knezević A.H., Sver L., Terzić S., Basić I. (2004). Immunomodulatory and antimetastatic action of propolis and related polyphenolic compounds. J. Ethnopharmacol..

[18] Machado J.L., Assunção A.K., da Silva M.C., Dos Reis A.S., Costa G.C., Arruda Dde S., Rocha B.A., Vaz M.M., Paes A.M., Guerra R.N. (2012). Brazilian green propolis: Anti-inflammatory property by an immunomodulatory activity. Evid. Based Complement. Alternat Med..

[19] Miguel M.G., Nunes S., Dandlen S.A., Cavaco A.M., Antunes M.D. (2010). Phenols and antioxidant activity of hydro-alcoholic extracts of propolis from Algarve, South of Portugal. Food Chem. Toxicol..

[20] Isla M.I., Nieva Moreno M.I., Sampietro A.R., Vattuone M.A. (2001). Antioxidant activity of Argentine propolis extracts. J. Ethnopharmacol..

[21] Sulaiman G.M., Sammarrae K.W.A., Ad’hiah A.H., Zucchetti M., Frapolli R., Bello E., Erba E., D’Incalci M., Bagnati R. (2011). Chemical characterization of Iraqi propolis samples and assessing their antioxidant potentials. Food Chem. Toxicol..

[22] De Araújo Pereira R.R., Godoy J.S.R., Svidzinski T.I.S., Bruschi M.L. (2013). Preparation and Characterization of Mucoadhesive Thermoresponsive Systems Containing Propolis for the Treatment of Vulvovaginal Candidiasis. J. Pharm. Sci..

[23] Bruschi M., Dota K., Consolaro M., Svidzinski T. (2011). Antifungal Activity of Brazilian Propolis Microparticles against Yeasts Isolated from Vulvovaginal Candidiasis. Evid. Based Complement. Alternat Med..

[24] Bonfim A.P., Sakita K.M., Faria D.R., Arita G.S., Vendramini F., Capoci I.R.G., Braga A.G., Dos Santos R.S., Bruschi M.L., Becker T.C.A. (2020). Preclinical approaches in vulvovaginal candidiasis treatment with mucoadhesive thermoresponsive systems containing propolis. PLoS ONE.

[25] Imhof M., Lipovac M., Kurz C., Barta J., Verhoeven H.C., Huber J.C. (2005). Propolis solution for the treatment of chronic vaginitis. Int. J. Gynaecol. Obstet..

[26] Pairazaman A.T.E., Pinto J.D.C., Chávez B.A., Apac G.L., Sandoval C.B.H., Quispe F.M.M., Meza V.A.J.C., Fretell W.G.I., Loyola G.A.R. (2022). Antifungal activity of propolis extract against *Candida albicans* in patients with vulvovaginal candidiasis. F1000Research.

[27] Sobel J.D. (2016). Recurrent vulvovaginal candidiasis. Am. J. Obstet. Gynecol..

[28] Denning D.W., Kneale M., Sobel J.D., Rautemaa-Richardson R. (2018). Global burden of recurrent vulvovaginal candidiasis: A systematic review. Lancet Infect. Dis..

[29] Nie J., Chen D., Ye J., Lu Y., Dai Z. (2021). Optimization and kinetic modeling of ultrasonic-assisted extraction of fucoxanthin from edible brown algae *Sargassum fusiforme* using green solvents. Ultrason. Sonochem..

[30] Wen C., Zhang J., Zhang H., Dzah C.S., Zandile M., Duan Y., Ma H., Luo X. (2018). Advances in ultrasound assisted extraction of bioactive compounds from cash crops—A review. Ultrason. Sonochem..

[31] Toma M., Vinatoru M., Paniwnyk L., Mason T.J. (2001). Investigation of the effects of ultrasound on vegetal tissues during solvent extraction. Ultrason. Sonochem..

[32] Shahidi F., Zhong Y. (2015). Measurement of antioxidant activity. J. Funct. Foods.

[33] Rodrigues F., Palmeira-de-Oliveira A., das Neves J., Sarmento B., Amaral M.H., Oliveira M.B. (2013). *Medicago* spp. extracts as promising ingredients for skin care products. Ind. Crops Prod..

[34] Krisch J., Ördögh L., Galgóczy L., Papp T., Vágvölgyi C. (2009). Anticandidal effect of berry juices and extracts from Ribes species. Open Life Sci..

[35] Gallucci M.N., Carezzano M.E., Oliva M., Demo M.S., Pizzolitto R.P., Zunino M.P., Zygadlo J.A., Dambolena J.S. (2014). In vitro activity of natural phenolic compounds against fluconazole-resistant Candida species: A quantitative structure–activity relationship analysis. J. Appl. Microbiol..

[36] Silva J.C., Rodrigues S., Feás X., Estevinho L.M. (2012). Antimicrobial activity, phenolic profile and role in the inflammation of propolis. Food Chem. Toxicol..

[37] Lagouri V., Prasianaki D., Krysta F. (2014). Antioxidant properties and phenolic composition of Greek propolis extracts. Int. J. Food Prop..

[38] Vongsak B., Kongkiatpaiboon S., Jaisamut S., Machana S., Pattarapanich C. (2015). In vitro alpha glucosidase inhibition and free-radical scavenging activity of propolis from Thai stingless bees in mangosteen orchard. Rev. Bras. Farmacogn..

[39] Choi S.J., Shimomura K., Kumazawa S., Ahn M.-R. (2013). Antioxidant properties and phenolic composition of propolis from diverse geographic regions in Korea. Food Sci. Technol. Res..

[40] Özkök A., Keskin M., Tanuğur Samancı A.E., Yorulmaz Önder E., Takma Ç. (2021). Determination of antioxidant activity and phenolic compounds for basic standardization of Turkish propolis. Appl. Biol. Chem..

[41] Ou S., Kwok K.C. (2004). Ferulic acid: Pharmaceutical functions, preparation and applications in foods. J. Sci. Food Agric..

[42] Tai A., Sawano T., Ito H. (2012). Antioxidative properties of vanillic acid esters in multiple antioxidant assays. Biosci. Biotechnol. Biochem..

[43] Mo F., Zhang P., Li Q., Yang X., Ma J., Zhang J. (2022). Development and Evaluation of a Film Forming System Containing Myricetin and Miconazole Nitrate for Preventing Candida albicans Catheter-Related Infection. Microb. Drug Resist..

[44] Lee H.-S., Kim Y. (2022). Myricetin disturbs the cell wall integrity and increases the membrane permeability of *Candida albicans*. J. Microbiol. Biotechnol..

[45] Hirasawa M., Takada K. (2004). Multiple effects of green tea catechin on the antifungal activity of antimycotics against *Candida albicans*. J. Antimicrob. Chemother..

[46] Almeida D., Pinto D., Santos J., Vinha A.F., Palmeira J., Ferreira H.N., Rodrigues F., Oliveira M.B.P.P. (2018). Hardy kiwifruit leaves (*Actinidia arguta*): An extraordinary source of value-added compounds for food industry. Food Chem..

[47] Bonfim-Mendonca Pde S., Ratti B.A., Godoy Jda S., Negri M., Lima N.C., Fiorini A., Hatanaka E., Consolaro M.E., de Oliveira Silva S., Svidzinski T.I. (2014). beta-Glucan induces reactive oxygen species production in human neutrophils to improve the killing of Candida albicans and Candida glabrata isolates from vulvovaginal candidiasis. PLoS ONE.

[48] Dantas Ada S., Day A., Ikeh M., Kos I., Achan B., Quinn J. (2015). Oxidative stress responses in the human fungal pathogen, Candida albicans. Biomolecules.

[49] Chen L., Wang F., Qu S., He X., Zhu Y., Zhou Y., Yang K., Li Y.X., Liu M., Peng X. (2022). Therapeutic potential of perillaldehyde in ameliorating vulvovaginal candidiasis by reducing vaginal oxidative stress and apoptosis. Antioxidants.

[50] de Francisco L., Pinto D., Rosseto H., Toledo L., Santos R., Tobaldini-Valério F., Svidzinski T., Bruschi M., Sarmento B., Oliveira M.B.P.P. (2018). Evaluation of radical scavenging activity, intestinal cell viability and antifungal activity of Brazilian propolis by-product. Food Res. Int..

[51] Zduńska K., Dana A., Kolodziejczak A., Rotsztejn H. (2018). Antioxidant properties of ferulic acid and its possible application. Skin. Pharmacol. Physiol..

[52] Yan-Chun Z., Rong-Liang Z. (1991). Phenolic compounds and an analog as superoxide anion scavengers and anti oxidants. Biochem. Pharmacol..

[53] Majer P., Neugart S., Krumbein A., Schreiner M., Hideg É. (2014). Singlet oxygen scavenging by leaf flavonoids contributes to sunlight acclimation in Tilia platyphyllos. Environ. Exp. Bot..

[54] Hu J., Calomme M., Lasure A., De Bruyne T., Pieters L., Vlietinck A., Vanden Berghe D. (1995). Structure-activity relationship of flavonoids with superoxide scavenging activity. Biol. Trace Elem. Res..

[55] Touzani S., Imtara H., Katekhaye S., Mechchate H., Ouassou H., Alqahtani A.S., Noman O.M., Nasr F.A., Fearnley H., Fearnley J. (2021). Determination of phenolic compounds in various propolis samples collected from an African and an Asian region and their impact on antioxidant and antibacterial activities. Molecules.

[56] Sobel J.D., Sobel R. (2018). Current treatment options for vulvovaginal candidiasis caused by azole-resistant Candida species. Expert. Opin. Pharmacother..

[57] Tobaldini-Valerio F.K., Bonfim-Mendonça P.S., Rosseto H.C., Bruschi M.L., Henriques M., Negri M., Silva S., Svidzinski T.I. (2016). Propolis: A potential natural product to fight Candida species infections. Future Microbiol..

[58] Duarte M.C.T., Figueira G.M., Sartoratto A., Rehder V.L.G., Delarmelina C. (2005). Anti-Candida activity of Brazilian medicinal plants. J. Ethnopharmacol..

[59] Facchinatto W.M., Galante J., Mesquita L., Silva D.S., dos Santos D.M., Moraes T.B., Campana-Filho S.P., Colnago L.A., Sarmento B., das Neves J. (2021). Clotrimazole-loaded N-(2-hydroxy)-propyl-3-trimethylammonium, O-palmitoyl chitosan nanoparticles for topical treatment of vulvovaginal candidiasis. Acta Biomater..

[60] das Neves J., Sarmento B. (2015). Precise engineering of dapivirine-loaded nanoparticles for the development of anti-HIV vaginal microbicides. Acta Biomater..

[61] das Neves J., Notario-Pérez F., Sarmento B. (2021). Women-specific routes of administration for drugs: A critical overview. Adv. Drug Deliv. Rev..

[62] Banskota A.H., Tezuka Y., Prasain J.K., Matsushige K., Saiki I., Kadota S. (1998). Chemical constituents of Brazilian propolis and their cytotoxic activities. J. Nat. Prod..

[63] Bonamigo T., Campos J.F., Oliveira A.S., Torquato H.F.V., Balestieri J.B.P., Cardoso C.A.L., Paredes-Gamero E.J., de Picoli Souza K., Dos Santos E.L. (2017). Antioxidant and cytotoxic activity of propolis of *Plebeia droryana* and *Apis mellifera* (Hymenoptera, Apidae) from the Brazilian Cerrado biome. PLoS ONE.

[64] Campoccia D., Ravaioli S., Santi S., Mariani V., Santarcangelo C., De Filippis A., Montanaro L., Arciola C.R., Daglia M. (2021). Exploring the anticancer effects of standardized extracts of poplar-type propolis: In vitro cytotoxicity toward cancer and normal cell lines. Biomed. Pharmacother..

[65] Cavalaro R.I., da Cruz R.G., Dupont S., de Moura Bell J.M.L.N., Vieira T.M.F.d.S. (2019). In vitro and in vivo antioxidant properties of bioactive compounds from green propolis obtained by ultrasound-assisted extraction. Food Chem. X.

[66] Pinto D., Braga N., Rodrigues F., Oliveira M.B. (2017). Castanea sativa Bur: An Undervalued By-Product but a Promising Cosmetic Ingredient. Cosmetics.

[67] Benzie I.F.F., Strain J.J. (1999). Ferric reducing/antioxidant power assay: Direct measure of total antioxidant activity of biological fluids and modified version for simultaneous measurement of total antioxidant power and ascorbic acid concentration. Methods in Enzymology.

[68] Re R., Pellegrini N., Proteggente A., Pannala A., Yang M., Rice-Evans C. (1999). Antioxidant activity applying an improved ABTS radical cation decolorization assay. Free Radic. Biol. Med..

[69] Moreira M.M., Barroso M.F., Boeykens A., Withouck H., Morais S., Delerue-Matos C. (2017). Valorization of apple tree wood residues by polyphenols extraction: Comparison between conventional and microwave-assisted extraction. Ind. Crops Prod..

[70] Gomes A., Fernandes E., Silva A.M.S., Santos C.M.M., Pinto D.C.G.A., Cavaleiro J.A.S., Lima J.L.F.C. (2007). 2-Styrylchromones: Novel strong scavengers of reactive oxygen and nitrogen species. Bioorg Med. Chem..

[71] (2017). Reference CLSI, Method for broth dilution antifungal susceptibility testing of yeasts. CLSI Standard M27.

[72] Präbst K., Engelhardt H., Ringgeler S., Hübner H. (2017). Basic colorimetric proliferation assays: MTT, WST, and resazurin. Methods Mol. Biol..

[73] Faria M.J., Machado R., Ribeiro A., Gonçalves H., Real Oliveira M.E.C.D., Viseu T., das Neves J., Lúcio M. (2019). Rational Development of Liposomal Hydrogels: A Strategy for Topical Vaginal Antiretroviral Drug Delivery in the Context of HIV Prevention. Pharmaceutics.

[74] Notario-Pérez F., Galante J., Martín-Illana A., Cazorla-Luna R., Sarmento B., Ruiz-Caro R., das Neves J., Veiga M.-D. (2021). Development of pH-sensitive vaginal films based on methacrylate copolymers for topical HIV-1 pre-exposure prophylaxis. Acta Biomat..

